# From Obscurity To Spotlight: Staphylococcus lugdunensis-Induced Infective Endocarditis, a Profound Case Unraveling

**DOI:** 10.7759/cureus.44685

**Published:** 2023-09-04

**Authors:** Tracy-Ann Poyser, Arthur Dilibe, Collie Shaw, Courtney M Hicks, Ethan Munzinger, David E Martin

**Affiliations:** 1 Internal Medicine, Unity Health - White County Medical Center, Searcy, USA; 2 Internal Medicine, East Carolina University Health, Greenville, USA; 3 Infectious Diseases, Unity Health - White County Medical Center, Searcy, USA; 4 Interventional Cardiology, Unity Health - White County Medical Center, Searcy, USA; 5 Graduate Medical Education, Unity Health - White County Medical Center, Searcy, USA

**Keywords:** cardiology, mitral valve replacement, coagulase- negative staphylococcus, infective endocarditis, staphylococcus lugdunensis

## Abstract

Infective endocarditis (IE) represents a grave infection characterized by endocardial inflammation and valve impairment due to diverse pathogens. *Staphylococcus lugdunensis*, a coagulase-negative organism, has garnered increasing recognition as a significant etiological agent of IE. This bacterium is renowned for its aggressive tissue infections encompassing bone and joint, bloodstream, and IE sites. Particularly noteworthy is the rapid devastation and abscess formation it induces on heart valves, resulting in elevated mortality rates. The pathogen's affinity for von Willebrand factor facilitates its attachment to cardiac valves and blood vessels, thereby exacerbating its virulence. This abstract provides a comprehensive overview of *S. lugdunensis*-triggered IE. We present a compelling case involving a 66-year-old female afflicted by IE attributed to this microorganism, illuminating the clinical manifestations and challenges linked to the ailment. Moreover, we scrutinize previously reported instances of *S. lugdunensis*-related IE spanning from 1993 to 2022, accentuating the escalating importance of this pathogen in disease causality. The deleterious consequences of *S. lugdunensis*-induced IE emanate from its distinctive clinical attributes, necessitating tailored diagnostic strategies and treatment considerations. Given the gravity and swift progression of the infection, healthcare professionals play a pivotal role in administering timely and efficacious management for afflicted patients. Further research is imperative to enhance diagnostic modalities and explore therapeutic approaches aimed at effectively combating this formidable and life-threatening infection.

## Introduction

*Staphylococcus lugdunensis* is a coagulase-negative staphylococcus (CoNS) organism characterized by its beta hemolysis on blood agar. This non-motile, facultative anaerobe was first elucidated in 1988. It stands distinct among CoNS due to its unique ability to cause aggressive native valve infective endocarditis (IE) [1]. While conventionally perceived as a skin commensal, this microorganism has evolved into a clinically significant pathogen that can induce severe infections, including IE. Its ability to swiftly erode valves and provoke abscess formation hinges on its interaction with the von Willebrand factor. This peculiar trait endows it with the capability to adhere to cardiac valves and blood vessel walls, enhancing its virulence akin to Staphylococcus aureus, bolstered by its gene-encoded protein implicated in iron acquisition from hemoglobin.

IE is a grave infectious ailment characterized by inflammation of the endocardium and heart valves, originating from pathogen colonization of cardiac structures. Native valve infection primarily emanates from organisms like streptococci, enterococci, *S. aureus*, and HACEK organisms (*Haemophilus* species, *Aggregatibacter* species, *Cardiobacterium hominis*, *Eikenella corrodens*, and *Kingella kingae*), gaining entry through damaged or anomalous heart valves. Typically sourced from the oral cavity, skin, or GI tract, these organisms are propelled into the bloodstream during dental procedures, surgeries, injections, or daily activities like tooth brushing. This infection, if untreated, is universally fatal [2].

Staphylococcus lugdunensis-induced IE, although infrequent (representing 1.1% of cases), presents diagnostic challenges and mimics other endocarditis-causing agents. Its tendency to form biofilms on cardiac tissues and provoke an inflammatory response makes diagnosis reliant on clinical observations, imaging studies, and microbiological identification. This ailment is linked to significant morbidity and mortality, necessitating swift diagnosis, effective treatment, and timely recognition to optimize outcomes. Management requires a multi-pronged approach, often including surgical intervention due to valvular damage, abscess formation, and persistent infection despite antibiotic treatment [3,4].

This paper comprehensively delves into *S. lugdunensis*-induced IE. We aim to augment healthcare professionals' understanding and awareness by elucidating clinical manifestations and challenges, thereby facilitating early identification and prompt treatment.

## Case presentation

**Figure 1:**
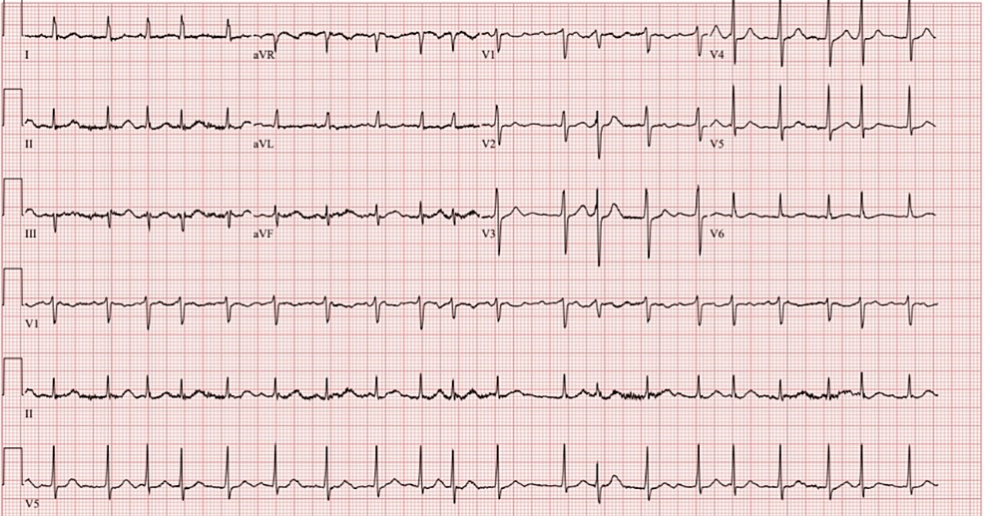
EKG on arrival showing atrial fibrillation with RVR. EKG: Electrocardiogram; RVR: Rapid ventricular response.

A 66-year-old female with a pertinent history of infiltrating ductal carcinoma of the breast and infusion port placement presented to the ED with altered mental status that had been ongoing for the past week. It was reported that the patient had recently been on a Caribbean cruise when she began experiencing fatigue, altered mental status, nausea, vomiting, diarrhea, and decreased oral intake. Her mental status deteriorated further upon returning from her cruise, prompting her family to bring her to the hospital. Collateral information from her relatives indicated no recent head trauma or falls. Upon arrival at the hospital, her vital signs were as follows: temperature of 39.2°C, blood pressure of 143/65 mmHg, heart rate of 135 beats per minute (bpm), respiratory rate of 24 breaths per minute, and oxygen saturation of 96% on ambient air. On examination, she appeared confused, ill, and in mild distress. Bilateral crackles were heard on auscultation. Her cardiovascular exam revealed tachycardia with an irregularly irregular rhythm, but no new murmurs were detected. Her abdomen was non-tender and exhibited normal bowel sounds. No focal deficit was observed on neurological examination, but the patient continued to have a waxing and waning mentation. On further evaluation with an electrocardiogram (EKG), the patient had atrial fibrillation with rapid ventricular response (RVR) (Figure 1).

Initial laboratory results (shown in Tables 1-3) were pertinent for leukocytosis with neutrophilic predominance, normocytic anemia, elevated brain natriuretic peptide (BNP), mild hyponatremia, hypokalemia, and an arterial blood gas (ABG) concerning for respiratory alkalosis.

**Table 1:** Showing the initial complete blood count on presentation. MCV: Mean corpuscular volume; ESR: Erythrocyte sedimentation rate; CRP: C-reactive protein.

Complete Blood Count (CBC)		
Variable	Results on Arrival	Reference range
WBC Count	14.6	4.50-11.00 k/uL
RBC Count	3.17	4.40-5.90 M/uL: male; 3.80-5.20 M/uL: female
Hemoglobin	11.4	13.0-18.0 g/dL: male; 12.0-16.0 g/dL: female
Hematocrit	27.6	40.0-52.0% - M; 35.0-47.0 % - F
MCV	87.2	80.0-100.0 fL
Platelet Count	153	150-440 k/uL
Absolute Neutrophil Count	11.68	1.80-7.70 k/uL
ESR	54	<20 mm/h
CRP	210	<5.0 mg/L

**Table 2:** Showing the initial basic metabolic panel upon presentation.

Basic Metabolic Panel (BMP)		
Variable	Results on Arrival	Reference Range
Sodium	130	136-145 mEq/L
Potassium	3.2	3.4-4.4 mEq/L
Chloride	96	98-107 mEq/L
Bicarbonate	23	22-29 mEq/L
Anion Gap	11	8-12 mEq/L
BUN	22	8-26 mg/dL
Creatinine	1.01	0.72-1.25 mg/dL
Calcium	9.8	8.4-10.2 mg/dL
Phosphorus	2.4	2.3-4.7 mg/dL
Magnesium	1.9	1.6-2.6 mg/dL
Lactic acid	1.5	0.5-2.0 mmol/L
Brain Natriuretic Peptide (BNP)	662	<=100 pg/mL

**Table 3:** Showing the initial arterial blood gas (ABG) results upon presentation.

Arterial Blood Gas (ABG)		
Variable	Result	Reference Range
pH	7.49	7.35-7.45
PCO2	31	35-45 mm Hg
PO2	74	60-100 mm Hg
HCO3	23	22-28 mEq/L

**Figure 2:**
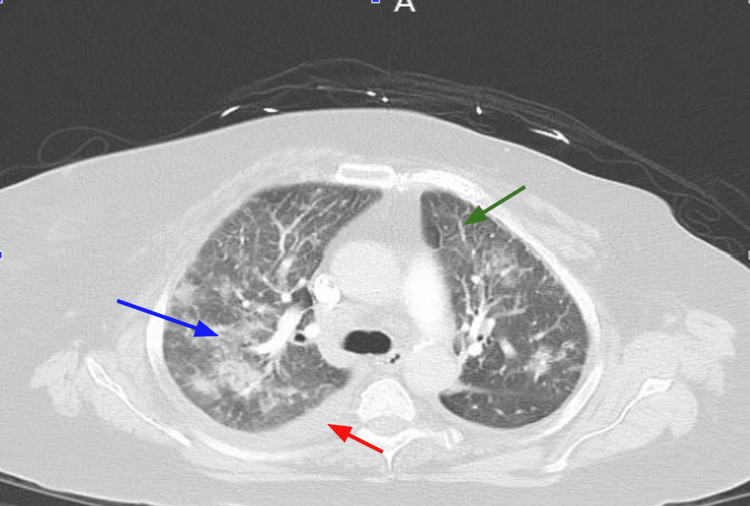
CTA pulmonary showing patchy peribronchovascular opacification (blue arrow), pleural effusion (red arrow), and smooth septal thickening (green arrow).

**Figure 3:**
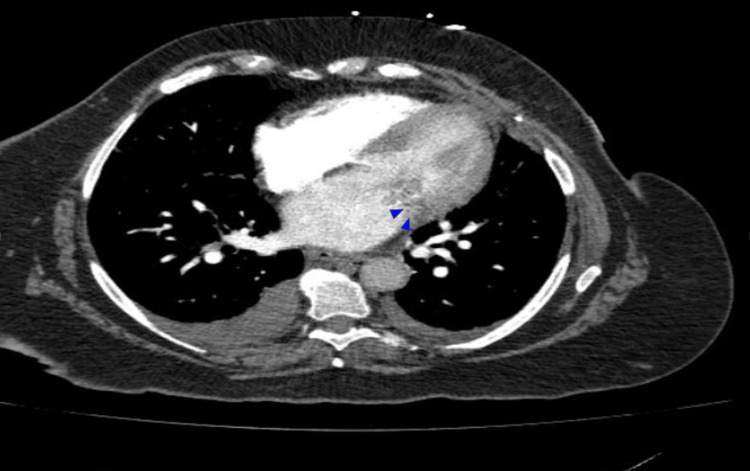
CTA pulmonary showing soft tissue density thickening along the left side of the mitral valve region (blue arrowheads) with some small internal calcifications. There was no pericardial fluid observed.

Given her fever, tachypnea, tachycardia, leukocytosis, a Systemic Inflammatory Response Syndrome (SIRS) score of 4, and a quick Sequential Organ Failure Assessment (qSOFA) score of 2, she was preliminarily assessed with suspicion for sepsis. Blood culture samples were collected, and she was initiated on IV fluid resuscitation and empiric antimicrobial therapy with IV vancomycin and IV ceftriaxone. A septic workup was commenced to identify a potential source, including urinalysis, lactate levels, and imaging of her head, chest, and abdomen. Urinalysis was generally unremarkable, and lactate levels were within normal limits. CT head without contrast did not show any acute intracranial abnormalities that might explain her encephalopathy. CT abdomen and pelvis showed previous Roux-en-Y procedure and no acute abnormalities. Computed tomography angiography (CTA) pulmonary showed multiple noncalcified nodules throughout both lungs measuring up to 7 mm in diameter, bilateral pleural effusions prominent on the right, peribronchial opacifications (Figure 2), and a soft tissue density (Figure 3) around the mitral valve that was concerning for vegetations. 

**Figure 4:**
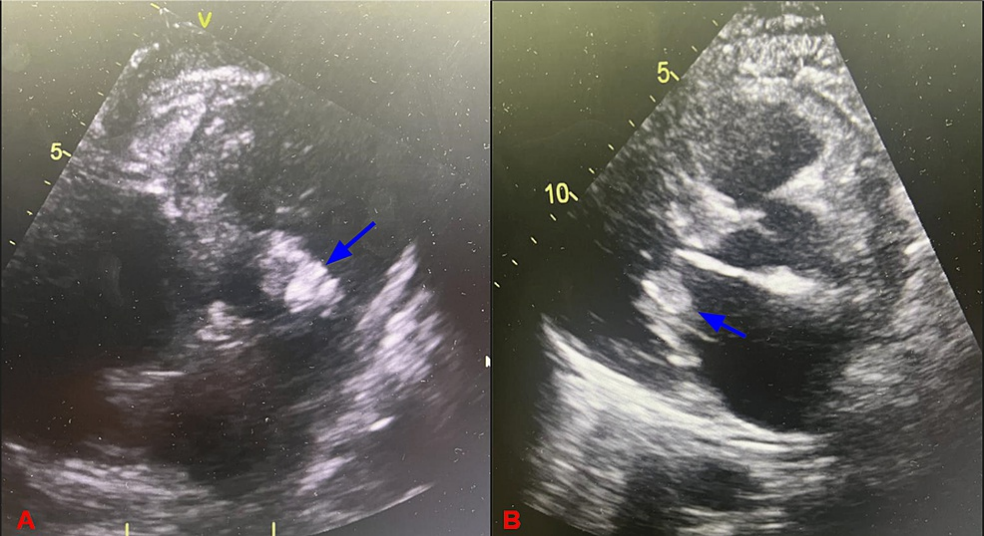
Echocardiographic image showing left-sided pleural effusion and mobile vegetation (blue arrows) on the posterior mitral leaflet, measuring 3.0 x 1.9 cm.

Due to new-onset atrial fibrillation with RVR and possible IE, cardiology was consulted, and the recommendation was made to start the patient on metoprolol tartrate for rate control and therapeutic enoxaparin. A transthoracic echocardiogram (TTE) was completed, which showed mobile vegetation on the posterior mitral leaflet, measuring 3.0 x 1.9 cm, moderate-to-severe mitral regurgitation, a severely dilated left atrium, and an ejection fraction (EF) of 60-65% (Figure 4).

The patient subsequently became hypotensive with a blood pressure of 87/54 mmHg and a mean arterial pressure (MAP) of 51. She was also found to have a lactate of 3.9, non-specific EKG changes, and an elevated troponin of 1.39. She was subsequently transferred to the cardiac intensive care unit (CICU) for further management of undifferentiated shock and non-ST-elevation myocardial infarction (NSTEMI). Empiric antimicrobial coverage was continued, and she was started on vasopressors. A further review of her medical history revealed no prior structural heart conditions. However, it was noted that she had an infusion-A-port replaced in 2016 and hardware in place from a previous right knee procedure. The blood culture report returned positive for methicillin-sensitive S*. lugdunensis*. Following the recommendations of our infectious disease specialists, we discontinued vancomycin and ceftriaxone and initiated treatment with cefazolin, administering 2 grams IV every 8 hours. Given the positive blood culture and echocardiographic evidence of vegetation, the patient met the diagnostic (modified Duke) criteria for definite IE caused by *S. lugdunensis*. Cardiology recommended that the patient get a heart catheterization to further evaluate her coronary arteries. However, she was deemed too hemodynamically unstable for the procedure. Her respiratory status continued to worsen with increasing oxygen requirements, and she was subsequently placed on Continuous Positive Airway Pressure (CPAP). A repeat physical examination revealed a new mid-diastolic rumble heard at the apex; auscultation of the lungs was pertinent for diffuse crackles bilaterally. Chest X-ray showed worsening pulmonary edema bilaterally, and the patient was treated with a single dose of IV furosemide 40 mg. Interdisciplinary consultation recommended that the patient would benefit from an urgent surgical valve repair/replacement. An intra-aortic balloon pump (IABP) was placed, and the patient was transferred to a tertiary care facility for cardio-thoracic surgery to evaluate for urgent mitral valve repair. Her mitral valve was replaced with a prosthetic valve at the tertiary facility.

## Discussion

*Staphylococcus lugdunensis* is a Gram-positive, catalase-positive CoNS. Despite being a CoNS, *S. lugdunensis* exhibits a virulence profile akin to *S. aureus* [5], contributing to its propensity to cause endovascular infections. Historically considered a commensal of the skin, *S. lugdunensis* has gained prominence as a clinically relevant pathogen capable of causing severe infections, including IE. The increasing incidence of *S. lugdunensis* IE has prompted the need for a detailed understanding of its clinical implications, microbiological characteristics, and management. 

A study by Petti CA et al. reported that *S. lugdunensis* was the second most common pathogen, behind *S. epidermidis*, in IE resulting from CoNS [6]. The ability of the microorganism to produce biofilms enhances its resistance to host defenses and antimicrobial therapy. Risk factors, such as prosthetic heart valves, intravascular devices, underlying valvular diseases, and prosthetic joint hardware, predispose patients to *S. lugdunensis* IE. Additionally, certain patient populations, such as immunocompromised individuals and those with IV drug use, are at higher risk of infection. A review of our patient's past medical history suggests that her immunocompromised state (history of breast cancer), her right knee prosthetic joint hardware, and an indwelling access port all predisposed her to this virulent pathogen.

S*taphylococcus lugdunensis* is often misidentified as other CoNS species during routine laboratory testing. This misidentification can delay appropriate treatment as different CoNS species may have different antibiotic susceptibilities and clinical implications. To overcome these diagnostic challenges, it is essential for healthcare providers to be aware of the unique characteristics of *S. lugdunensis* and consider it as a potential pathogen in cases of suspected endocarditis. Molecular methods like polymerase chain reaction (PCR) and matrix-assisted laser desorption/ionization time-of-flight mass spectrometry (MALDI-TOF MS) can help in accurate identification [7].

In a retrospective review by Liu PY et al., *S. lugdunensis* IE predominantly occurred in patients above 50 years of age, most of whom acquired the infection in the community with unidentified entry sites. Patients diagnosed with *S. lugdunensis* IE were notably older than those with *S. aureus*-induced IE, with an average age of 73 compared to 66 years. Most isolated *S. lugdunensis* strains were sensitive to penicillin, and the infection often affected left-side valves, particularly the mitral or aortic valves, and sometimes both simultaneously. This contrasts with other CoNS, which typically invade prosthetic valves and are often resistant to penicillin [8].

Previously published studies that compare the in-hospital mortality rate of IE caused by *S. lugdunensis* to rates from *S. aureus* and other CoNS have suggested notable differences. Specifically, the all-cause mortality at 30 days was higher in the *S. lugdunensis* group (20%, n = 6) compared to the other CoNS group (7%, n = 17) and the *S. aureus* group (9%, n = 166), with a statistical significance of p = 0.016. These findings highlight that, in certain instances, *S. lugdunensis* can lead to a more aggressive form of IE and poorer outcomes. This underscores the importance of early identification and the need for prompt medical management [9].

The clinical presentation of *S. lugdunensis* IE is similar to other forms of IE and includes fever, new or changing heart murmurs, embolic phenomena, and constitutional symptoms [5-9]. Blood cultures remain the cornerstone for diagnosis, and repeated culture attempts may be necessary due to the organism's slow growth rate. Furthermore, advanced imaging technique, such as transesophageal echocardiography, aid in detecting valve vegetations and complications like abscess formation. 

The management of *S. lugdunensis* IE is challenging due to its inherent resistance to certain antibiotics and the propensity for aggressive disease progression. The organism is generally susceptible to many antibiotics, but the specific choice of drugs and the duration of treatment should be guided by the results of antimicrobial susceptibility testing. Beta-lactam antibiotics are often used as the first-line treatment for susceptible strains. In some cases, surgical intervention may become necessary, especially if heart valve destruction, abscess formation, or persistent infection are present [9,10,11].

## Conclusions

*Staphylococcus lugdunensis* is an increasingly important pathogen responsible for IE. Understanding its unique clinical characteristics, diagnostic challenges, and treatment considerations is crucial for healthcare professionals in order to provide timely and effective management for affected patients. Further research is needed to improve diagnostic methods and explore novel therapeutic strategies to combat this challenging infection effectively.
